# The Role of Galectins in Chronic Lung Allograft Dysfunction

**DOI:** 10.1007/s00408-021-00449-3

**Published:** 2021-05-03

**Authors:** Miriana d’Alessandro, Laura Bergantini, Antonella Fossi, Elda De Vita, Felice Perillo, Luca Luzzi, Piero Paladini, Piersante Sestini, Paola Rottoli, Elena Bargagli, David Bennett

**Affiliations:** 1 Respiratory Diseases Unit, Department of Medical Sciences, University Hospital of Siena (Azienda Ospedaliera Universitaria Senese, AOUS), Viale Bracci, 53100 Siena, Italy; 2 Thoracic Surgery Unit, Cardio-Thoracic and Vascular Department, University Hospital of Siena (Azienda Ospedaliera Universitaria Senese, AOUS), Siena, Italy

**Keywords:** Galectin-1, Galectin-3, Galectin-9, Lung transplantation, Chronic lung allograft dysfunction, Biomarkers

## Abstract

**Background:**

Galectins are proteins that bind β-galactosides such as *N*-acetyllactosamine present in N-linked and O-linked glycoproteins and that seem to be implicated in inflammatory and immune responses as well as fibrotic mechanisms. This preliminary study investigated serum galectins as clinical biomarkers in lung transplant patients with chronic lung allograft dysfunction (CLAD), phenotype bronchiolitis obliterans syndrome (BOS).

**Materials and Methods:**

Nineteen lung transplant patients [median age (IQR), 55 (45–62) years; 53% males] were enrolled in the study. Peripheral blood concentrations of galectins-1, 3 and 9 were determined with commercial ELISA kits.

**Results:**

Galectin-1 concentrations were higher in BOS than in stable LTX patients (*p* = 0.0394). In logistic regression analysis, testing BOS group as dependent variable with Gal-1 and 3 as independent variables, area under the receiver operating characteristics (AUROC) curve was 98.9% (NPV 90% and PPV 88.9%, *p* = 0.0003). With the stable LTX group as dependent variable and Gal-1, 3 and 9 as independent variables, AUROC was 92.6% (NPV 100% and PPV 90%, *p* = 0.0023). In stable patients were observed an inverse correlation of Gal-3 with DLCO% and KCO%, and between Gal-9 and KCO%.

**Conclusion:**

Galectins-1, 3 and 9 are possible clinical biomarkers in lung transplant patients with diagnostic and prognostic meaning. These molecules may be directly implicated in the pathological mechanisms of BOS. The hypothesis that they could be new therapeutic targets in BOS patients is intriguing and also worth exploring.

## Introduction

Lung transplant (LTX) is a lifesaving treatment option for patients with end-stage lung diseases, including emphysema, cystic fibrosis, pulmonary fibrosis and pulmonary arterial hypertension, unresponsive to maximal medical therapy or for whom no effective medical or other surgical alternative exists [1–4]. Long-term survival after LTX is still challenging and chronic lung allograft dysfunction (CLAD) remains a leading cause of death [5]. Bronchiolitis obliterans syndrome (BOS) is the most common form of CLAD and appears to begin with an inflammatory stage, characterized by lymphocytic peribronchiolar infiltrates, and then progresses to a fibroproliferative stage with proliferation of fibroblasts and accumulation of collagen under the bronchiolar epithelium, leading to obliteration of the airway lumen [6]. A restrictive form of CLAD, termed restrictive allograft syndrome (RAS), has also recently been recognized [7].

Galectins (Gal) are proteins that bind β-galactosides, such as *N*-acetyllactosamine, present in N-linked and O-linked glycoproteins. Galectin structure features one or two carbohydrate recognition domains, on the basis of which they are classified as prototype galectins (e.g. Gal-1), chimera galectins (e.g. Gal-3) and tandem-repeat galectins (e.g. Gal-9). Galectins have numerous biological functions: they participate in cross-talk and maintenance of cell architecture, facilitating interactions between cells and components of the extracellular matrix [8]. These cell–cell and cell–matrix interactions, as well as signalling pathways on the cell surface, influence and modulate cell functions, such as inflammatory and immune responses [9]. Depending on the type of inflammatory stimulus, the microenvironment and the target cells involved, these proteins may be associated with pro- or anti-inflammatory responses [10]. Galectins are also implicated in pulmonary fibrosis, where their inhibition may be useful in therapeutic management [11–14]. Some reports show that malignant transformation in human cancer patients is often associated with altered galectin expression [15]. Galectins play a crucial role in the homeostasis of inflammation through their apoptotic effect on activated leukocytes [11, 16]. Release of Gals-1 and 3 initiates an inflammatory response in several ways, including binding of neutrophils to the endothelium, trafficking of neutrophils through the extracellular matrix bound to laminins and fibronectin, and acting as chemotactic agents towards the site of inflammation [12, 13]. Once neutrophils have been trafficked to the scene, galectins-1 and 3 then contribute to the respiratory burst that enables neutrophils to destroy cell contents or invading pathogens [8, 17]. Some data in kidney and liver transplantation have been published regarding their potential role in graft tolerance [18, 19], but no data is available on the role of galectins in LTX patients. In the present study our aim was to explore the potential of serum concentrations of galectins-1, 3 and 9 as clinical biomarkers in lung transplant patients with BOS.

## Materials and Methods

### Study Population

Nineteen lung transplant patients [median age (IQR), 55 (45–62) years; 53% males] monitored at the Siena Regional Referral Centre for Sarcoidosis and other Interstitial Lung Diseases were enrolled in the study. Nine patients [median age (IQR), 51 (49–63) years; 33% males] were considered stable (CLAD-free), whereas the other ten patients [median age (IQR), 58 (37–62) years; 70% males] had CLAD (BOS phenotype). A healthy control group (HC) was also included (*n* = 9; median age (IQR), 45 (35–54) years; 33% males).

CLAD was defined in presence of a substantial and persistent decline (≥ 20%) in measured FEV_1_ value from the reference (baseline) value. The baseline value was computed as the mean of the best two postoperative FEV_1_ measurements (taken > 3 weeks apart). BOS phenotype was assumed in case of a fall in FEV_1_ ≥ 20% (compared with baseline) in presence of an obstruction pattern at spirometry (FEV_1_/FVC < 0.7) without persistent radiologic pulmonary opacities (parenchymal opacities and/or increasing pleural thickening consistent with a diagnosis of pulmonary and/or pleural fibrosis and likely to cause a restrictive physiology, rather than the airway-based changes consistent with bronchiectasis) [6]. All included patients met BOS diagnostic criteria, patients with RAS were excluded.

All clinical and functional data was collected retrospectively and entered in a specific database. Patients gave their written informed consent to participation in the study, which was approved by our local ethics committee (Respir1, Prot n 15,732, 16 September 2019).

### Lung Function Tests

Lung function measurements were performed according to ATS/ERS standard procedure [20], using a Jaeger body plethysmograph with corrections for temperature and barometric pressure: forced expiratory volume in the first second (FEV1), forced vital capacity (FVC), total lung capacity (TLC), residual volume (RV), diffusing capacity of the lung for carbon monoxide (DLCO) and carbon monoxide transfer coefficient (KCO) for alveolar volume. All parameters were expressed as percentages of predicted values. They were recorded at sampling time and after LTX in order to establish CLAD stage.

### Galectin Assay

Peripheral blood concentrations of galectins-1, 3 and 9 were determined with the Human Galectin quantikine ELISA kit (R&D System, Minneapolis, USA). Nine serum samples were also collected from the healthy volunteers and assayed. The serum concentrations of Gal-1 [mean (IQR), 18.1 (13.9–28.2) ng/ml], Gal-3 [mean (IQR), 6.73 (2.40–15.7) ng/ml] and Gal-9 [mean (IQR), 7 (3.1–10.4) ng/ml] of controls were in line with those indicated by the kit manufacturer [21].

### Statistical Analysis

The data did not show a normal distribution. The Mann–Whitney test was used for comparison of two variables, while non-parametric one-way ANOVA (Kruskal–Wallis test) and the Dunn test were used for multiple comparisons. The Chi-squared test was used for categorical variables, as appropriate. According to the presence of BOS phenotype, our population were divided in CLAD-free and CLAD-BOS group.

We also performed a logistic regression with HC as dependent variable against CLAD-BOS (or CLAD-free) to assess the potential of the serum galectins for differential diagnosis between the groups. Sensitivity, specificity, and positive and negative predicted values (PPV and NPV, respectively) were calculated for cut-offs of the different variables. Correlations between variables were studied by Spearman correlation and linear regression. A p-value less than 0.05 was considered statistically significant. Statistical analysis and graphic representation of the data was performed with GraphPad Prism 8.0 software. Unsupervised and supervised Principal Component Analysis (PCA) was performed by ClustVis (https://biit.cs.ut.ee/clustvis).

## Results

The main characteristics of our population, including serum concentrations of galectins-1, 3 and 9 and lung function parameters, are reported in Table 1.

**Table 1:** Main characteristic of population including age, gender, smoking habit, underling lung diseases and comorbidities, galectins and lung function test parameters

Parameters	CLAD-free (*n* = 9)	CLAD-BOS (*n* = 10)	Healthy controls (*n* = 9)	
Age	49 (51–63)	37 (58–62)	25 (30–58)	
Gender (male/female)	3/6	7/3	2/7	
Smoking habit (never/former)	6/3	4/6	4/5	
Diagnosis				
Pulmonary fibrosis	4 (44.5%)	3 (30%)		0.51
COPD	2 (22.3%)	3 (30%)		0.70
Cystic fibrosis	2 (22.3%)	4 (40%)		0.40
Other diagnosis	–	–		–
Comorbidities				
Diabetes mellitus	2 (22.3%)	5 (50%)		0.21
Arterial hypertension	1 (11.2%)	3 (30%)		0.31
Hypercholesterolemia	2 (22.3%)	4 (40%)		0.40
Osteoporosis	4 (44.5%)	7 (70%)		0.58
Galectins concentration				
Gal-1 (mg/dl)	22.7 (18.7–29.4)	29.9 (25.6–58.1)	13.1 (11.6–16.6)	0.0026
Gal-3 (mg/dl)	10.7 (4.6–17.6)	12.1 (9.4–16.9)	6.2 (3–8.1)	0.0154
Gal-9 (mg/dl)	8.6 (7.3–15.6)	12.3 (9.3–19.3)	3.8 (2.1–6.1)	0.0003
LFTs				
FVC (ml)	2460 (2150–3445)	2765 (2273–2945)		> 0.999
FVC%	90 (71–99)	69 (61–85)		0.0877
FEV1 (ml)	2090 (1710–2580)	1800 (1303–2138)		0.2359
FEV1 (%)	87 (66–91)	57 (42–71)		0.0079
FEV1/VC	81 (73–87)	64 (62–70)		0.0293
DLCO (%)	69 (55–78)	62 (54–85)		0.7243
KCO (%)	82 (72–95)	92 (71–108)		0.6304

All data were expressed as median and Interquartile range

**Fig. 1:**
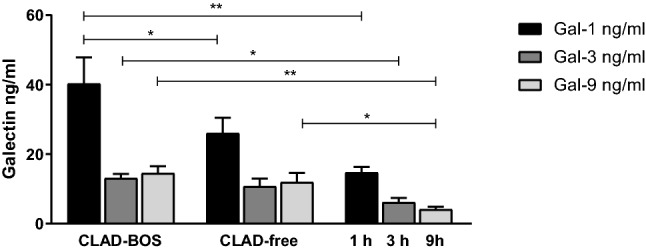
Galectin concentrations in LTX patients, including CLAD and stable patients and healthy controls. *p < 0.05; **p < 0.005

Galectin-1, 3 and 9 concentrations were significantly higher in CLAD-BOS patients than in HC (*p* = 0.0018, *p* = 0.0122 and *p* = 0.0003, respectively). Galectin-1 concentrations were higher in CLAD-BOS than in CLAD-free patients (*p* = 0.0394); Gal-9 concentrations were higher in the CLAD-free group than in the HC group (*p* = 0.0231) (Table 1, Fig. 1). No difference in Galectin-1, 3 and 9 concentrations accordingly underling lung disease or other basal characteristics were observed.

**Fig. 2:**
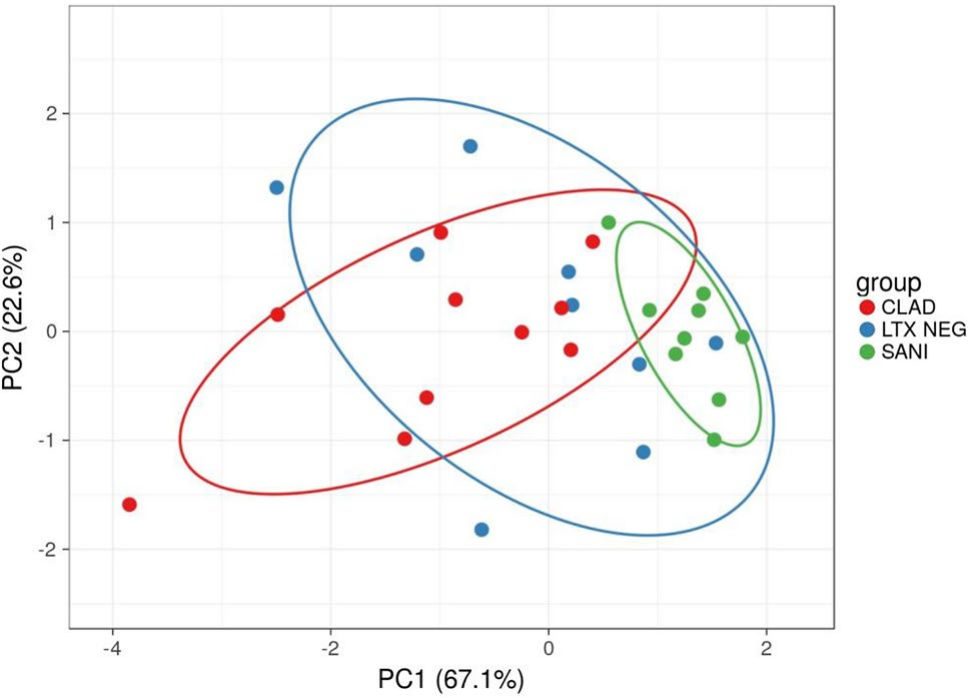
Unit variance scaling is applied to rows; SVD with imputation is used to calculate principal components. *X* and *Y* axis show principal component 1 and principal component 2 that explain 67.1% and 22.6% of the total variance, respectively. Prediction ellipses are such that with probability 0.95, a new observation from the same group will fall inside the ellipse. *N* = 28 data points. *LTX neg* stable LTX; *CLAD* chronic allograft lung dysfunction

PCA analysis was performed with the results obtained for serum concentrations of galectins. The eigenvalue percentage significance of the PCs was 99.9%. Intraclass dispersion occurred mainly along the first and second PCs. The three groups separated along the first PC (Fig. 2).

**Fig. 3:**
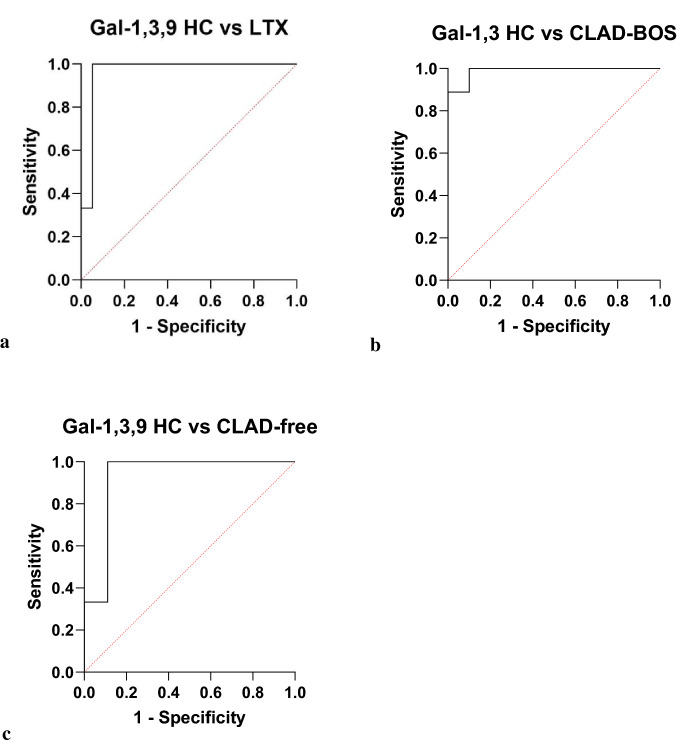
**a** HC group as dependent variable and Gal-1,3,9 as independent variables, areas under the ROC (AUROC) curve of 96.5% (95% CI 89–100, NPV 100% and PPV 90%, *p* < 0.0001). **b** CLAD group as dependent variable with Gal-1 and 3 as independent variables, AUROC was 98.9% (95% CI 95–100, NPV 90% and PPV 88.9%, *p* = 0.0003). **c** stable LTX group as dependent variable with Gal-1,3,9 as independent variable, AUROC was 92.6% (95% CI 78–100, NPV 100% and PPV 90%, *p* = 0.0023)

Testing the HC group as dependent variable by logistic regression, with Gal-1, 3 and 9 as independent variables, we obtained an area under the ROC (AUROC) curve of 96.5% (95% CI 89–100, NPV 100% and PPV 90%, *p* < 0.0001) (Fig. 3a). When we tested the CLAD-BOS group as dependent variable with Gal-1 and 3 as independent variables, AUROC was 98.9% (95% CI 95–100, NPV 90% and PPV 88.9%, *p* = 0.0003) (Fig. 3b). With the CLAD-free group as dependent variable and Gal-1, 3 and 9 as independent variables, AUROC was 92.6% (95% CI 78–100, NPV 100% and PPV 90%, *p* = 0.0023) (Fig. 3c).

A significant inverse correlation of Gal-3 with DLCO% and KCO% was observed among CLAD-free, while Gal-9 showed inverse correlations with KCO% among stable patients, and with measured FVC in CLAD-BOS patients (the latter with borderline significance, but with a good Rho coefficient: − 0.714, *p* = 0.058) (Table 2). No significant correlations were observed between Gal-1 concentrations and lung function parameters (Table 2).

**Table 2:** Main correlations between Galectin concentrations and LFT parameters

CLAD-free		Rho coefficient	*P* value	CLAD-BOS		Rho coefficient	*P* value
Gal-1	FVC%	0.217	0.581	Gal-1	FVC%	− 0.275	0.507
	FVC(ml)	0.400	0.291		FVC (ml)	− 0.190	0.665
	FEV1%	0.517	0.162		FEV1%	− 0.238	0.582
	FEV1(ml)	0.550	0.133		FEV(ml)	0.095	0.840
	DLCO%	− 0.117	0.776		DLCO%	0.300	0.683
	KCO%	− 0.367	0.336		KCO%	0.600	0.350
	RV%	0.453	0.882		RV%	0.369	0.332
	TLC%	0.190	0.772		TLC%	0.309	0.730
Gal-3	FVC%	− 0.117	0.776	Gal-3	FVC%	0.323	0.434
	FVC (ml)	− 0.083	0.843		FVC(ml)	0.262	0.536
	FEV1%	− 0.450	0.230		FEV1%	0.571	0.151
	FEV1(ml)	− 0.333	0.385		FEV1(ml)	0.619	0.115
	DLCO%	− 0.800	0.014		DLCO%	0.300	0.683
	KCO%	− 0.683	0.050		KCO%	0.100	0.950
	RV%	0.112	0.213		RV%	0.192	0.782
	TLC%	0.310	0.221		TLC%	0.886	0.033
Gal-9	FVC%	0.133	0.744	Gal-9	FVC%	− 0.623	0.105
	FVC (ml)	0.033	0.948		FVC(ml)	− 0.714	0.058
	FEV1%	− 0.183	0.644		FEV1%	− 0.643	0.096
	FEV1(ml)	− 0.167	0.678		FEV1(ml)	− 0.595	0.132
	DLCO%	− 0.567	0.121		DLCO%	− 0.100	0.950
	KCO%	− 0.800	0.014		KCO%	− 0.200	0.783
	RV%	0.683	0.05		RV%	0.143	0.867
	TLC%	0.700	0.043		TLC%	0.324	0.584

## Discussion

BOS is the most frequent presenting phenotype of CLAD. It is characterized by bronchiole thickening and obstruction due to injury and inflammation of epithelial cells and small airway subepithelial structures, leading to fibroproliferation and aberrant tissue repair [22]. Since pathogenesis is very complex, there are currently no biomarkers for early diagnosis of BOS or with clear clinical prognostic significance. The present exploratory study evaluated serum concentrations of galectins-1, 3 and 9 for the first time in LTX recipients, with and without BOS, and in healthy controls to assess their potential role as clinical biomarkers.

Gal-1 has been demonstrated to play a negative prognostic role in several conditions, including cancer and fibrotic processes [23, 24]. In transplantation, Gal-1 has shown to participate to hepatic graft tolerance and has been postulated it can be implicated in the inhibition of dendritic cells-mediated allogeneic T cell stimulation and survival and Th1/Th17 production [19, 25, 26]. It is also implicated in the dysregulation of the ERK/MAPK pathway [12] and plays its major function of immunomodulation determining the reduction of INF-gamma and inducing IL5 and IL10 production under inflammatory conditions [17] thus participating to neutrophil homeostasis [27]. While in a rat model of acute kidney rejection Gal-1 showed a potential protective role [28], more recently it was found increased in the glomeruli of kidney transplant patients with antibody-mediated rejection and proposed as potential therapeutic target to prevent extracellular matrix remodelling in such condition [29]. In the present study, higher serum concentrations of Gal-1 were observed in CLAD patients with BOS than in CLAD-free patients and healthy controls, which could be related to the inflammatory status of patients in the chronic rejection group. BAL neutrophilia is a common finding in BOS patients that can predict response to azithromycin therapy [30, 31]. Galectin-1 can positively or negatively modulate the effector functions of neutrophils according to cell activation stage [32] and its increased concentration might reflect the activation of neutrophils in BOS. Some earlier studies reported anti-inflammatory action of Gal-1, initially by modulation of pro-inflammatory cytokine release and PMN migration through an imbalance of adhesion molecule expression, and later by promoting monocyte-macrophage recruitment [33]. Galectin-1 can also activate neutrophils through CD43, depending on the environment in which it acts [34]. Our results highlight the potential of Gal-1 as a clinical biomarker of BOS and suggest that it plays an as yet undefined part in the pathogenic mechanisms of BOS.

In the present study, higher serum concentrations of Gal-3 were observed in CLAD patients with BOS than in healthy controls, although its levels in BOS patients did not differ significantly with respect to those of CLAD-free patients. Galectin-3 concentrations were correlated with DLCO% and KCO% in CLAD-free patients. Elevated Gal-3 concentrations are known to be correlated with various inflammatory and fibrotic conditions, including idiopathic pulmonary fibrosis, cardiovascular disease, autoimmune diseases, cancer and osteoarthritis, but no data is yet available on lung transplant patients [35, 36]. Galectin-3 seems to play a role in renal Ischemic-reperfusion injury involving the secretion of macrophage-related chemokine, pro-inflammatory cytokines and ROS production [37].

Our limited number of patients does not allow us a safe interpretation of this result; however, the possible association with some pre-CLAD lung pathological process within the graft is interesting and need further exploration.

Galectin-9 was first identified as a chemoattractant and activation factor of eosinophils in vitro and in vivo [38]. It modulates different biological functions including cell aggregation and adhesion, as well as apoptosis of tumour cells. Few papers reported the prognostic role of Gal-9 in different organ transplantations in which Gal-9 expression after allo-hematopoietic stem cell and liver transplantation is a potential prognostic biomarker of acute rejection [39–41]. Moreover, some studies reported the association of Galectin-9 and the balance between infection/rejection in kidney transplantation [42] and it has been postulated its immunoregulatory role during the ongoing cytotoxic response [43]. In the present study, we recorded higher serum concentrations of Gal-9 in LTX patients than in healthy controls. Our findings can be traced back to fibroproliferation and aberrant tissue repair after injury and inflammation of small airway subepithelial structures in BOS patients. We also found inverse correlations between Gal-9 and KCO% in CLAD-free patients and between Gal-9 and measured FVC in CLAD-BOS, suggesting that this protein could be a negative prognostic marker in LTX recipients.

The limits of the present study were its monocentric and retrospective nature and small statistical sample. However, as an exploratory study into the role of galectins in lung transplant patients, it could pave the way for new research into their clinical and pathogenic participation in BOS. No specific hypothesis on their mechanistic role in lung transplantation can be made at this point; however, our study shines a first spotlight on galectins in chronic lung allograft dysfunction suggesting their potentiality as marker for homeostatic mechanisms of the graft that worth to investigate.

In conclusion, this is the first study to evaluate galectins concentrations in serum of LTX recipients. Our results reveal overexpression of these molecules in CLAD patients with BOS phenotype correlated with several respiratory function parameters. Galectins-1, 3 and 9 are possible clinical diagnostic and prognostic biomarkers, worthy of further study in larger populations in order to clarify their role in lung transplant. These molecules may be directly implicated in the pathological mechanisms of BOS. The hypothesis that they could be new therapeutic targets in BOS patients is intriguing and also worth exploring.
